# Current methodologies of greenspace exposure and mental health research—a scoping review

**DOI:** 10.3389/fpubh.2024.1360134

**Published:** 2024-03-05

**Authors:** Julius Freymueller, Hannah-Lea Schmid, Ben Senkler, Susanne Lopez Lumbi, Stefan Zerbe, Claudia Hornberg, Timothy McCall

**Affiliations:** ^1^Medical School OWL, Department of Sustainable Environmental Health Sciences, Bielefeld University, Bielefeld, Germany; ^2^Faculty of Agricultural, Environmental and Food Sciences, Free University of Bozen-Bolzano, Bolzano, Italy; ^3^Institute of Geography, University of Hildesheim, Hildesheim, Germany; ^4^School of Public Health, Department of Environment and Health, Bielefeld University, Bielefeld, Germany

**Keywords:** greenspace, mental health, methodology, natural outdoor environments, nature, well-being, urban green, public health

## Abstract

**Introduction:**

Greenspaces can provide an important resource for human mental health. A growing body of literature investigates the interaction and the influence of diverse greenspace exposures. In order to gain a comprehensive understanding of the complex connection between greenspace and mental health, a variety of perspectives and methodological combinations are needed. The aim of this review is to assess the current methodologies researching greenspace and mental health.

**Methods:**

A scoping review was conducted. Four electronic databases (Pubmed, Embase, PsycInfo, Web of Science) were searched for relevant studies. A wide range of greenspace and mental health keywords were included to provide a comprehensive representation of the body of research. Relevant information on publication characteristics, types of greenspaces, mental health outcomes, and measurements of greenspace exposure and mental health was extracted and assessed.

**Results:**

338 studies were included. The included studies encompassed a multitude of methods, as well as outcomes for both greenspace and mental health. 28 combinations were found between seven categories each for greenspace and mental health assessment. Some pairings such as geoinformation systems for greenspace assessment and questionnaires investigating mental health were used much more frequently than others, implying possible research gaps. Furthermore, we identified problems and inconsistences in reporting of greenspace types and mental health outcomes.

**Discussion:**

The identified methodological variety is a potential for researching the complex connections between greenspace and mental health. Commonly used combinations can provide important insights. However, future research needs to emphasize other perspectives in order to understand how to create living environments with mental health benefits. For this purpose, interdisciplinary research is necessary.

## 1 Introduction

Nature in general and particularly greenspaces in urban environments can provide a wide range of resources and services for human populations (1, 2). Greenspaces can have a positive impact on physical as well as mental health (1, 3–5). Especially in areas with a high population density such as cities and megacities, greenspaces can provide an important resource for population health if they can be accessed by a great number of residents (6). Markevych et al. (7) propose three domains of pathways, which link greenspace to positive health outcomes: 1. The reduction of harm (*mitigation*) describes the potential protection from environmental stressors such as air pollutants, environmental noise or urban heat islands through greenspace. Furthermore, 2. greenspaces can provide opportunities for the building of capacities (*instoration*). Greenspace can offer places for social contacts as well as possibilities for physical activities (1, 5). Lastly, Markevych et al. (7) describe 3. the restoring of capacities (*restoration*) as a domain pathway through which greenspaces can contribute to psychological restoration.

All three of these pathways are relevant for research regarding the influence of greenspaces on mental health, which is described with a wide variety of outcomes such as psychological disorders, mood or restoration. The WHO (8) defines mental health as “a state of well-being in which every individual realizes his or her own potential, can cope with the normal stresses of life, can work productively and fruitfully, and is able to make a contribution to her or his community.” Psychological theories linked to the restoration domain can provide a theoretical basis for mental health benefits. The Attention Restoration Theory (ART) (9) and the Stress Recovery Theory (SRT) (10, 11) are often taken into account as a theoretical framework for this interaction (6, 7). The ART states that natural environments can provide a higher fascination or the sense of being away from undesirable aspects of everyday life compared to urban environments and can provide effortless attention which in turn leads to restoration (9). The SRT (10, 11) describes natural environments with a high proportion of natural elements, like vegetation or open water bodies as less threatening compared to urban environments from a psycho-evolutionary point of view. As a result, according to the SRT natural environments contribute to stress reduction and restoration (10, 11).

The contribution of greenspaces to the improvement of mental health has been investigated in a growing number of publications (12–14). Especially in urban areas, where the risk for mental illnesses is generally higher compared to rural areas greenspaces contribute to better health outcomes (15). Scientific reviews mostly display a consistent association between greenspace and mental health in the general population as well as emotional and behavioral well-being in children (16–18), although some find limited or inadequate evidence (19).

While the number of publications regarding greenspace has risen (13, 14), definitions of greenspace often vary across disciplines, leading to different understandings of greenspace and difficulties in making comparisons (4). A review regarding greenspace definitions in scientific studies identified two main interpretations of greenspace, but also underlines that no single definition of greenspace is generally applicable. Rather the definition used should be meaningful in the context of the study (20). In addition to the variation in definitions, discrepancies in the measurement of greenspaces limit the comparability of publications (7, 21). This is particularly relevant as the characteristics and quality of greenspaces can be important factors for their health impact (1, 12). Trees, for example, might be more beneficial to memory or mental health in comparison to other types of green elements (22–24) and formal, well-kept greenspaces could potentially provide a greater health benefit than other (e.g., wilder, more natural) greenspaces (25). Relevant health factors of greenspaces such as biodiversity, greenspace types, and the quality of greenspaces are often not included in measurements, for example in the frequently used Normalized Density Vegetation Index (NDVI) (7, 24, 26–28). Quality assessment tools can provide options for the inclusion of greenspace quality in scientific investigations (29). Furthermore, methods employed to measure the exposure to greenspace in the everyday life of participants through, for instance, wearable technologies with integrated Global Positioning Systems (GPS) can enable researchers to assess these exposures more accurately (7, 21).

Additionally, the measurements used for the assessment of mental health differ in the context of greenspace research. Questionnaires, epidemiological measurements, and biomarkers have been used in previous research (7). Similar to the methods for the assessment of greenspace, these methods have distinct potentials and drawbacks that should be considered in the design of studies. In their scoping review regarding the research of greenspace and mental well-being, Wendelboe-Nelson et al. (18) found a higher usage of self-developed questionnaires than validated tools. Since validation is an important characteristic of high-quality health assessment tools (30) this use of unvalidated questionnaires can pose an issue for the reporting of mental health.

Consequently, the definitions and methods employed measuring greenspace as well as mental health and their combinations are essential for the interpretation of the findings, however this has not been studied in the detail yet. Several reviews exist regarding the research of greenspace and mental health outcomes. Some focused on specific characteristics of greenspaces such as their biodiversity (28), indoor plants (31) or trees (32), while others focused on certain populations such as children (16, 33) or the pathways between greenspaces and health (34). Similarly, the effects of the COVID 19-pandemic on the use patterns as well as the influence of greenspace regarding mental health have been investigated within existing reviews (35, 36). There are fewer reviews focusing on methods, and those that do are analyzed from either an ecological or health perspective or put their emphasis on specific methods, such as biopsychological health outcomes (6) or Geoinformation System (GIS)-based exposure measures (37). Another review investigated different perspectives of research on urban greenspaces on several health outcomes and how green places, a term emphasizing the personal bonding to and perceptions of greenspace, could be more beneficial for health than greenspace (38). Collins et al. (12) created a systematic map for the research regarding greenspace in the context of mental health. They identified categories for the investigation of experimental as well as observational studies. Wendelboe-Nelson et al. (18) also focused their review on the methods used in the impact of greenspace on mental health but mostly reported the mental health aspect and did not give a detailed account of combination of method usage.

To our knowledge, there is no quantitative overview of the vast range of methods employed in environmental mental health research. In particular, the assessment of both greenspace exposure and mental health measures and their combination in research has not been reviewed yet. Accordingly, this scoping review aims to answer the following questions:

- Which greenspace and mental health methods are used in scientific studies?
- How are different methods of greenspace exposure measurements and mental health outcomes linked in scientific studies?
- What are the research gaps resulting from the methodological combinations?

Based on our results, this review aims to provide practical recommendations for future research concerning the measurements of greenspace and mental health. To this end, both greenspace and mental health methods are assessed and categorized in the results. Furthermore, the greenspace types and mental health outcomes are categorized. The combinations of identified methods are displayed in the results as well as the distribution of virtual and real greenspace within the included studies. Finally, the research questions are discussed and conclusions are provided.

## 2 Materials and methods

The aim of the study was to give an overview of current methods used in the broad and heterogenous research area of greenspaces and mental health. Therefore, a scoping review was deemed the most suitable methodological choice in order to outline this broad field of research and identify research gaps (39, 40). Additionally, this review aimed to research methodologies regarding this field of research, while not investigating the evidence of individual studies (41). The Preferred Reporting Items for Systematic reviews and Meta-Analyses extension for Scoping Reviews (PRISMA-ScR) were utilized to report the methodological steps and the results within this scoping review (42, 43). After an extensive literature review in order to identify relevant key terms, the search string for the database search was created using a PEO framework, which includes the population, exposure and outcome. A PEO framework was chosen because the control category of the usually utilized PICO framework was not deemed applicable for the purpose of this review, and greenspace was categorized as an exposure rather than an intervention. As for the population category, several settings where greenspace can be applied were included, such as “urban,” “town” or “municipality.” The outcome category included a variety of mental health outcomes. The search string was applied to the electronic databases Medline (Pubmed), Embase, PsycInfo and Web of Science. The search took place on the 23rd of May 2022. The review was not pre-registered.

The inclusion and exclusion criteria used for study selection are shown in Table 1. To be included, studies had to examine urban and rural greenness as direct or indirect primary exposure. Furthermore, at least one mental health indicator had to be measured, e.g., cognition or stress. The studies had to focus their analysis mainly on adults and adolescents above the age of 12 years, in order to avoid redundancies with other reviews (16). Studies focusing on the effects of greenspaces on animals and plants were excluded. To reflect the currently used mental health and greenspace methods, only studies between 2017 and 2022 were included in this review. Additionally, the search was limited to studies conducted in English or German. If publications reported the findings of multiple experiments, only experiments relevant to this review were included and only unique accounts of relevant information were counted. Some of the included studies also analyzed other environmental exposures such as air pollution, noise or blue space. Due to the scope of this review on greenness and the great number of studies, these exposures were not examined but the relevant measurements of greenspace were included. In order to include a broad range of mental health outcomes a variety of different search terms such as stress, general mental health, cognitive function or mood were used. The search terms for each database are presented in Supplementary Table S1.

**Table 1:** Inclusion and exclusion criteria employed for the screening process.

Inclusion criteria	Exclusion criteria
Primary exposure measurement of urban and rural green exposure, directly or indirectly	Studies which exclusively analyzed different exposures, e.g., air pollution
Primary measurement of at least one mental health indicator	Additional therapeutic intervention, e.g., psychological counseling in greenspace
Studies including human subjects	Effects of greenspace on animals or plants
Mainly adolescents above the age of 12 and adults	Studies mainly regarding children under the age of 12
Publication between 2017 and 2022	Published before 2017
Publication in either English or German	
Study Design: Empirical studies and experimental studies, original paper	

As mentioned above, a wide range of definitions of greenspace has been previously applied. Taylor and Hochuli (20) identified two main definitions, the first greenspace definition referring to natural areas in general including areas of vegetation as well as bodies of water. The second definition describes greenspace as vegetated areas of open spaces in the urban environment. While blue spaces were not included within the search terms of this review, a broad range of greenspaces is included in order to gain a comprehensive understanding of the different types of greenspaces investigated in scientific publications.

The review process included several stages. Publications were independently reviewed by two researchers. First, a title screening was conducted in order to exclude studies not fitting for further screening by five authors (BS, JF, H-LS, SLL, TM). Studies that were included by one of the screeners were included in the next step. Second, an abstract screening was carried out in order to further limit the number of studies. Each publication was screened by two of the aforementioned five authors (BS, JF, H-LS, SLL, TM). Disagreement between the screening authors was resolved by a third opinion from another author. Due to the scope of this work on methodological approaches regarding connections between greenspace exposure and mental health outcomes, the next screening focused on the examination of the method section within the included studies. Two of the aforementioned five authors screened the methods of each study separately (BS, JF, H-LS, SLL, TM). Disagreement was resolved as described above. The WHO-5 questionnaire (44) was not included in this review as a relevant tool since it was considered as a measurement to assess the general well-being of participants rather than mental well-being as a mental health outcome.

After completion of the screening processes, a form for data-charting of relevant information was created via discussion between the authors (45). The relevant data was independently charted using a standardized extraction table by two authors (BS and JF). Data from 10% of the included studies were charted by both extracting authors to ensure compatibility of data-charting between the reviewers. The results of joined charting were discussed with another author (H-LS). Data was extracted regarding general information such as publication characteristics, e.g., the year of publication and country as well as information on the studied population. Specific information was charted for types of greenspaces, tools used for the measurement of greenspace exposure, mental health outcomes and the assessments used for mental health. The charted information is available in Supplementary Table S2.

In order to visualize the combinations of methods identified in the included studies, a categorization of the greenspace and mental health methods was necessary. Furthermore, greenspace types and mental health outcomes were categorized as an additional layer of information. The categories for greenspace types, greenspace measurements, mental health outcomes and the mental health measurements were iteratively synthesized from the results of the screening process, while previous research regarding greenspace and mental health was also used to improve the categorization (17, 21, 26, 29). The categories identified in this review are presented in the results. All authors were involved in the discussion of relevant categories.

As the systematical assessment of study quality is an optional criterion of scoping reviews according to the PRISMA-ScR (42) and the main focus of this review was to showcase the variety of methods used for the assessment of mental health and greenspace and not to evaluate the quality of individual studies in detail, no quality assessment was conducted in this review.

## 3 Results

**Figure 1:**
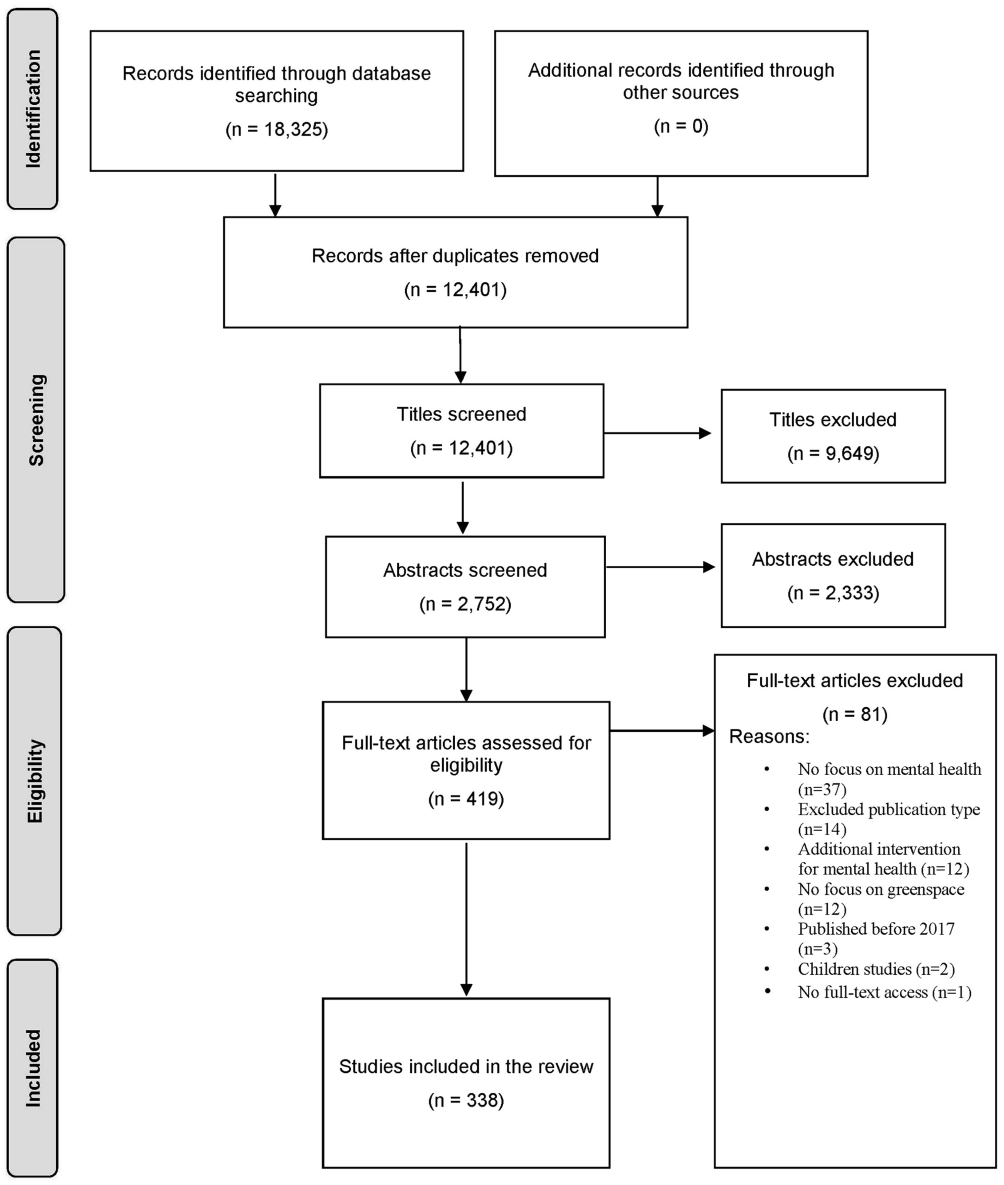
PRISMA flow diagram of included studies for this review.

This chapter presents the search results after the completed screening process. During the search in the four databases, 12,401 publications were identified after removing the duplicates. In the title screening, 9,649 studies were excluded. In the ensuing abstract screening, a further 2,333 studies were excluded. The remaining 419 full texts were then reviewed according to the inclusion and exclusion criteria. In the process, 81 studies were excluded, e.g., because they did not focus on mental health or greenspace or did not match included publication types. After the screening process a total of 338 studies were included in this review (*cf.* Figure 1).

The studies were conducted in different regions and countries. The greatest proportion of studies were conducted in China (*n* = 66). Furthermore, there were 46 studies conducted in the United States, followed by 19 studies in Australia.

In addition, different study designs were used which can be categorized as longitudinal, cross-sectional, experimental, qualitative, and mixed methods. Cross-sectional designs were the most common, with a total of 141 studies. The second most common design was experimental, with 118 fully experimental studies. Longitudinal designs were used in 54 publications. A mixed methods approach was applied by six studies and qualitative surveys were identified in 15 studies to define the relationships between green exposure and mental health.

The different populations in the study period can be divided into different categories (see Table 2). The majority of studies (*n* = 213) do not describe their populations further, classifying them either as park visitors, (healthy) adults, or simply as participants. As selection criteria are often not clear or not reported, this group is referred to as the “general population.” Furthermore, other studies do not draw a classic sample, but select data on the basis of administrative units (*n* = 7), so that individual residential districts or urban areas are examined. There are also demographic aspects, such as age, into which the study populations can be divided. Thus, there are studies of adolescents (*n* = 21), an even larger proportion of young adults/students (*n* = 65), and some studies of older adults (over 60 years; *n* = 23). In addition, there is a small number of studies that have included patients with different medical conditions (*n* = 12), such as schizophrenia, but also other physical conditions, such as pollen allergy.

**Table 2:** Distribution of population categories within the included studies.

Type of population	Number of counts in included studies
General (adult) population	213
Young adults/students	65
Older adults (over 60)	23
Adolescents	21
Patients (with medical condition)	12
Administrative units	7

Some studies (*n* = 3) investigated more than one type of population, which is why the total number of study populations differs from the number of included publications.

### 3.1 Greenspace types

This section provides a comprehensive overview regarding the research of different greenspace types within the included studies. The greenspace types are clustered in three spatially distinct ecological scales which encompass: ‘natural elements and species’, ‘land-use types and ecosystems’, and ‘landscapes’, the latter including patches and not specifically defined greenspaces (see Table 3). Regarding the spatial scales, most included studies focused on land-use types or landscapes instead of natural elements. 52 studies investigated two scales and five studies all three scales. Within the natural elements scale, trees (50%) and indoor plants (30%) were mainly investigated. Public parks (47%) and forests (34%) were assessed most often among the land-use types. Across all scales, urban green was the greenspace type mostly researched, making up 88% of the landscapes scale within the included studies.

**Table 3:** Spatial scales, corresponding greenspace types, and the number of papers assessing them.

Spatial scales	Greenspace types	Number of counts in included studies
Natural elements and species groups		20 (in total)
	Trees	10
	Indoor plants	6
	Grasses	3
	Ornamental plants	3
	Greening of buildings	2
	Shrubs	1
Land-use types and ecosystems		197 (in total)
	Public parks	92
	Forest, woodland	66
	Gardens	32
	Tree cover	26
	Grassland, meadow	19
	Roadside Greenery	11
	Shrubland	9
	Arable land	7
	Courtyard	2
	Informal greenspace	1
	Cemetery	1
Landscapes, including patches and non-specific greenspace		184 (in total)
	Urban Green	162
	Rural Greenspace	40

Some studies (*n* = 104) investigated more than one greenspace type, which is why the total number of types differs from the number of included publications.

Natural elements include single plant specimens and populations, such as trees [e.g., (46, 47)], indoor plants [e.g., (48, 49)], grass patches [e.g., (50)], or ornamental plants [e.g., (51)]. Natural elements were investigated 20 times, with 35% of studies applying virtual exposures.

Land-use types and ecosystems, respectively, refer to areas defined by their vegetation and land usage, in total 197 studies, 18% focused on virtual greenspaces. Land-use types and ecosystems comprise, e.g., public parks [e.g., (52–54)], forests [e.g., (55–57)], and gardens [e.g., (58–60)]. Among this category were also studies that, after closer inspection, were found to investigate blue spaces (*n* = 13). However, this was not sufficiently described by the authors and therefore could not be excluded in the eligibility step (Figure 1).

Landscapes can be defined as “spatially heterogeneous geographic areas characterized by diverse interacting patches or ecosystems” (61) and as “perceived by people, whose character is the result of the action and interaction of natural and/or human factors” (62). A total of 184 studies investigated landscapes, 9% of which used virtual landscapes. In our review, this category includes urban green landscapes or landscape patches [e.g., (22, 63, 64)] and rural greenspaces [e.g., (65–67)].

### 3.2 Greenspace measurements

Greenspace measurements describe the methodological approach regarding the assessment of greenspaces within the included studies. This review differentiates between seven methodological approaches to assess greenspace exposure, which are described further below. Table 4 displays the number of publications using the different methods. Some publications used multiple greenspace assessment methods. Accordingly, the count of greenspace exposure methods exceeds the number of included studies.

**Table 4:** Categories of greenspace assessment methods and their count within papers.

Greenspace assessment method	Number of counts in included studies
Geoinformation systems	145
Predefined through intervention	120
Self-reported quantitative	56
Expert assessment	21
Self-reported qualitative	18
Street view	8
Ecological momentary assessment	7

Some studies (*n* = 40) applied more than one greenspace assessment method, which is why the total number of methods differs from the number of included publications.

The category ‘Geoinformation systems’ (GIS) describes methods using the analysis of spatial data in order to assess greenspace exposure. Firstly, this includes vegetation indices such as the NDVI [e.g., (68–70)]. Secondly, GIS encompasses land-use data gathered via satellite data or from land registry offices and other sources, describing land-use types [e.g., (71–73)]. In both cases, studies mostly focused on the abundance or proximity of greenspaces (38). Of the included studies 145 studies used GIS, thus it was the most common of all greenspace assessment methods.

The category ‘Predefined through intervention’ includes studies that applied a predefined exposure, such as controlled activities, e.g., walking, viewing a particular scene or exercising, in order to measure the impact on mental health [e.g., (74–76)]. Additionally, this category encompasses studies using images, videos or virtual reality as a controlled greenspace exposure [e.g., (77–80)]. At least one predefined controlled exposure was employed in 120 publications.

‘Self-reported quantitative’ includes methods assessing the study population’s self-reported greenspace exposure, mostly via questionnaires. A variety of exposure characteristics, such as the frequency and duration of visits to greenspaces, the amount or quality of nearby greenspaces, as well as the proximity of nearby greenspaces, were reported in the included studies [e.g., (81–84)]. Self-reported quantitative measurements were applied as a greenspace assessment method in 56 publications.

The category ‘Expert assessment’ refers to studies in which the study area was either described and distinguished by researchers based on certain characteristics, such as naturalness, area size or biodiversity [e.g., (85–87)], or an assessment of certain greenspace indicators, such as greenspace quality, was performed by the researchers or other experts [e.g., (83, 88, 89)]. Expert assessments were included in 21 studies.

‘Self-reported qualitative’ (SR qualitative) refers to studies that assessed greenspace exposure through qualitative methods, such as semi-structured interviews, thematic writing or participatory methods [e.g., (90–93)]. These studies generated an in-depth insight in the greenspace exposure of the study population, which mostly consisted of fewer than 25 subjects. Methods with a qualitative exposure assessment were included in 18 studies.

‘Street view’ (SV) includes studies using images from an eye-level perspective gathered by street-view services in order to assess the study population’s greenspace exposure. This measurement can be employed via buffer areas around the study participant’s residence or in the neighborhood [e.g., (94–96)]. Another possibility to assess every day greenspace exposure is to measure the eye-level greenspace on routes which are regularly used by the individual person [e.g., (97, 98)]. SV was used in eight studies.

‘Ecological momentary assessment’ (EMA) describes the assessment of the momentary exposure of the study population combined with a mental health measurement at a certain time. These exposures can be self-reported by the participants [e.g., (99, 100)] or gathered via a combination of GPS signals and GIS or SV calculations [e.g., (101–105)]. This enables the researchers to collect data of every day greenspace exposure in relation to momentary mental health outcomes. In total, seven of the included publications employed EMA.

### 3.3 Mental health outcomes

This section provides an overview regarding the different mental health domains which were investigated by the included studies. This review differentiates a total of 10 different mental health outcome categories in the included studies, which are described below. All clustered outcomes and the number of counts are listed in Table 5.

**Table 5:** Categories of mental health outcomes and the corresponding number of papers assessing them.

Mental health outcome	Number of counts in included studies
Affect & mood	148
Stress	96
Mental disorder	90
Restoration	58
Mental health	57
Cognitive outcomes	43
Brain activity & structure	33
Mental well-being	32
Vitality	12
Miscellaneous	23

Some studies (*n* = 153) investigated more than one mental health outcome, which is why the total number of outcomes differs from the number of included publications.

‘Affect & mood’ was the most commonly found outcome category within the included studies (*n* = 148). This category describes different experiences of emotional states, feelings and mood. These studies can examine different affect states using a range of tools, such as affect with the Positive and Negative Affect Schedule (PANAS), which considers items such as active, distressed, proud or irritable, but also trait & state anxiety with the State Trait Anxiety Index [e.g., (74, 86)]. The latter includes 20 items regarding trait anxiety and 20 items for assessing state anxiety. Another frequently used tool was the Profile of Mood States (POMS), which assesses more transient states such as anger, vigor or fatigue [e.g., (104)].

The category ‘stress’ (*n* = 96) includes studies in which the psychological stress or tension of a person was measured. Methods used were mainly questionnaires with self-constructed items or validated instruments like the Perceived Stress Scale, which differentiates between low, moderate and high perceived stress, or objective measurements, e.g., cortisol levels [e.g., (106, 107)].

The ‘mental disorder’ category (*n* = 90) includes outcomes where the authors directly addressed mental illness. These were measured in different ways. On the one hand, some studies collected epidemiological disease figures from various registry data, such as health prescription rates of psychotropic medication [e.g., (108)]. On the other hand, studies utilized individual validated diagnostic survey instruments such as the Alzheimer’s Disease Assessment Scale (ADAS), which assesses the progression regarding symptoms of dementia [e.g., (109)].

‘Restoration’ includes 58 studies which measured with the effects of mental fatigue on concentration according to the ART. For example, questionnaires such as the Perceived Restorativeness Scale (PRS), that investigates the psychological aspects regarding restorative influences, were used to assess the restorative effects of exposure to greenery [e.g., (80)].

The category ‘mental health’ (*n* = 57) includes more general questions about mental health states that do not address a specific domain within mental health. In this category, questionnaires, such as the 12-item form of the General Health Questionnaire (GHQ-12), assessing, e.g., the ability to carry out every day functions, or the Short Form (SF-12), which measures for example general mental health, are used [e.g., (110, 111)].

‘Cognitive outcomes’ (*n* = 43) include all tests that measure domains such as perception, conceiving, remembering, reasoning, judging, imagining, and problem solving [e.g., (112)]. Different domains of memory were tested, for example, via the spatial working memory span task or the backwards digit span test [e.g., (49, 55)]. In order to measure cognitive outcomes, tools such as the Wechsler adult intelligence scale, that assesses cognitive abilities such as working memory via testing, were used [e.g., (113)].

The category ‘brain activity and structure’ (*n* = 33) includes studies that provide information about neuronal activity or morphological structure by using various imaging methods or the measurement of electrical neurophysiological impulses to find possible mechanisms for psychological outcomes. For example, magnetic resonance imaging (MRI) or Electroencephalogram (EEG) were used in the included studies [e.g., (105, 114)].

In addition, there is a sub-category similar to ‘mental health’ that examines ‘mental well-being’ (*n* = 32). These terms are often used interchangeable in policies and academic literature (115). Mental well-being seeks to cover aspects of affect as well as psychological functioning from both a hedonic as well as an eudaimonic perspective according to Tennant et al. (115). For the purpose of measuring mental well-being, tools such as the Short Warwick-Edinburgh Mental Well-being Scale (WEMWBS), which covers, e.g., eudemonic well-being and psychological functioning, were used [e.g., (116)].

The category ‘vitality’ (*n* = 12) mostly refers to outcomes that operationalize the state of feeling alive and awake, of having personal energy. This is measured with various scales and questionnaires, such as the Subjective Vitality Scales [e.g., (117)]. The two included scales cover ongoing individual vitality and state-based vitality.

The ‘miscellaneous’ category (*n* = 23) includes various items that did not seem to fit into any of the other categories, but still examined mental health. This includes outcomes such as sleepiness, smoking behavior or emotional eating patterns [e.g., (106, 118, 119)]. Some of these studies also used validated tools, such as the Karolinska sleepiness scale, which assesses sleepiness in regard to the psycho-physical state [e.g., (119)].

### 3.4 Mental health measuring methods

Regarding the possibilities for mental health assessment methodologies, seven categories were identified in this review. Table 6 displays the number of publications using the different methods. Some publications applied multiple mental health methods. Accordingly, the count of mental health methods exceeds the total number of studies.

**Table 6:** Mental health measuring methods and number of papers applying them.

Mental health method	Number of counts in included studies
Questionnaire	255
Physiological marker	62
Cognitive testing	39
Neurological indicator	29
Epidemiological measurement	27
Qualitative measurement	22
Behavior or facial expression	8

Some studies (*n* = 82) applied more than one mental health assessment method, which is why the total number of methods diverts from the number of included publications.

The category ‘Questionnaire’ includes validated questionnaires, such as the Mental Health Inventory (MHI-5), the GHQ-12 or the PANAS [e.g., (110, 120, 121)], as well as author-constructed questionnaires [e.g., (122, 123)]. These were applied for the measurement of various mental health outcomes. The outcomes were mostly self-reported by the participants, only few of the included studies had other groups, such as parents, answering a questionnaire about the participants [e.g., (124)]. 255 studies employed one or multiple questionnaires to assess mental health, the highest number of uses of any mental health method in this review.

Another category of methods assessing mental health are ‘physiological marker’, which for example encompasses blood pressure, heart rate variability, salivary cortisol or eye-tracking [e.g., (60, 123, 125, 126)]. These measurements were regularly used as physiological indicators for stress reactions or as restoration outcomes. A total of 62 studies utilizing physiological markers were included in the review.

The category ‘cognitive testing’ includes the controlled testing of mental health outcomes with a variety of tests, such as the Stroop test, the Sustained Attention to Response Test (SART) or the Mini-Mental State Examination [MMSE; e.g., (77, 127–129)]. These tests enabled the researchers to quantify the performance for certain cognitive outcomes or the onset of diseases, such as dementia. Within the included studies, tests measuring the cognitive functions of the study participants were utilized in a total of 39 studies.

‘Neurological indicator’ as a category includes methodologies that are used to quantify brain activity and certain brain structures. These indicators were measured for example via functional magnetic resonance imaging (fMRI), which is used to analyze brain activities via MRI or EEGs and employed mostly as proxies for mental health outcomes [e.g., (66, 105, 130, 131)]. Neurological indicators were used in 29 publications.

‘Epidemiological measurement’ encompasses studies using the incidence, medication sales, the length of stay in an health care institution or other epidemiological measurements to assess mental health [e.g., (89, 132–134)]. The studies included in this review only applied epidemiological measurements for mental health disorders as an outcome. Epidemiological measurements were employed 27 times in the included studies.

‘Qualitative measurement’ describes the measurement of mental health via qualitative methods, such as semi-structured interviews and focus groups [e.g., (135–137)] or qualitative participatory methods such as photovoice [e.g., (91, 138)]. These methods were mostly employed in order to assess the mental health effects of greenspaces regarding a certain study population, such as gardeners or residents/visitors of a predefined area [e.g., (58, 91, 139, 140)]. 22 studies used qualitative measurements.

The category ‘behavior or face recognition’ consists of two methods, which were used in 8 of the included studies. Firstly, it includes studies which used behavior screening, such as number of cigarettes smoked or food consumed, as a mental health outcome [e.g., (106, 118)]. Secondly, some studies utilized face recognition in order to assess the emotions of the people represented in pictures, mostly gathered from social media [e.g., (141–143)].

### 3.5 Combination of methods

**Figure 2:**
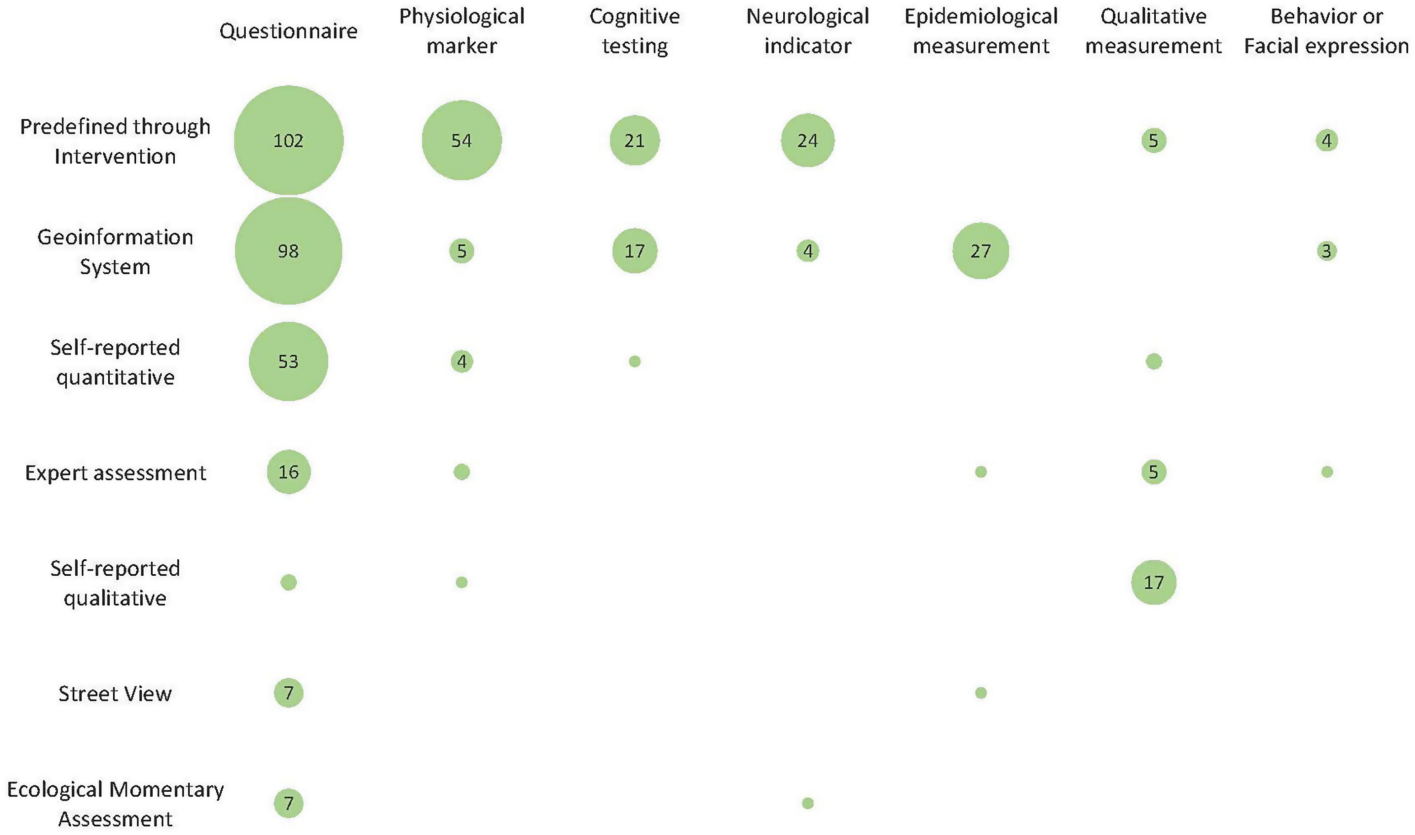
Usage of greenspace exposure measurements and measures assessing mental health. The size of the bubbles correlates to the number of studies using the corresponding combination.

This chapter combines the methodologies used to assess greenspace as well as mental health and describes the frequency of use of the combinations. Figure 2 displays the usage of greenspace exposure measurements as well as measures assessing mental health. The size of the bubbles relates to the number of times a certain combination was used in comparison to other combinations. Additionally, the count of each combination is shown inside the bubble except for combinations that were only found once or twice. Some studies used multiple methods in order to assess greenspace or mental health or both. Accordingly, the count of total combinations is larger than the number of studies included in the review. The total count of combinations identified is 484.

The count of studies using certain methodological combinations varies substantially between the individual combinations. While some combinations of methods were used in numerous studies, other pairings were not found at all within the included studies.

A high number of studies used a predefined exposure of greenspace in an interventional setting. 102 publications utilized a combination of predefined exposure of greenspace with questionnaires, which corresponds to the highest number of a single combination identified in this review. Physiological markers and predefined greenspaces were combined in 54 publications, by far the highest number of combinations between a greenspace exposure and physiological markers. Also, neurological indicators were used in 24 studies in a predefined setting which is the highest count of usage of this mental health measurement. Cognitive testing was applied in 21 studies. Qualitative measurement and behavior or facial expressions were combined five and four times with predefined greenspace exposure, respectively.

GIS was applied in combination with all of the mental health methods except for qualitative measurements. GIS was most often combined with questionnaires, identified in a total of 98 studies. Epidemiological measurements and GIS were employed in 27 studies. Publications using cognitive testing and GIS were found 17 times. Neurological indicators, physiological markers and behavior or facial expression were all employed five times or less with GIS.

Self-reported quantitative exposure of greenspace was used with questionnaires as a mental health assessment in 53 studies. Less frequently, self-reported quantitative exposure was employed with physiological markers (*n* = 4). Qualitative health measurements were used twice in combination with self-reported quantitative exposure (144, 145), while cognitive testing (146) was employed once in combination with self-reported quantitative greenspace measurements.

Expert assessments were most commonly employed with questionnaires, in total 16 times. Qualitative mental health measurements were used in five publications with expert assessments, while physiological marker (85, 147), epidemiological measurements (89) as well as behavior or facial expression (148) were employed in not more than two studies for the assessment of mental health in combination with expert assessments.

Self-reported qualitative assessment of greenspace was most frequently combined with the qualitative measurement of mental health, with 17 publications employing this combination. Two studies assessing greenspace via self-reported qualitative data used questionnaires for the assessment of mental health (149, 150) and one study included the measurement of physiological markers (151).

The assessment of greenspace via SV was combined with questionnaires seven times and only one study combined SV with epidemiological measurements (152).

EMA was most frequently utilized in combination with questionnaires as well, with seven studies using this combination. In one case an fMRI was employed as a neurological indicator in an EMA study (105).

### 3.6 Intervention studies

An additional dimension used to distinguish the included studies that predefined the exposure with greenspace in an intervention was the differentiation between real and virtual greenspaces. Real greenspaces include the exposure to real world, physical greenspace. Whereas, virtual greenspaces describe the exposure through photos or videos of greenspaces, as well as uses of virtual reality environments, and pre-recorded sounds of greenspaces. Half of the studies with predefined exposure used real greenspace (*n* = 103) and half investigated virtual greenspace (*n* = 102). The distribution is depicted in Table 7.

**Table 7:** Distribution of virtual and real greenspace for mental health assessment methods in studies with predefined exposure.

	Questionnaire	Qualitative measurement	Cognitive testing	Neurological indicator	Physiological marker	Behavior or face recognition	Total
Real	49	4	10	13	26	–	102
Virtual	50	1	11	11	26	4	103
Real and virtual	3	–	–	–	1	–	4
Total	102	5	21	24	53	4	209

Some studies utilizing predefined exposures (*n* = 67) applied more than one mental health assessment method, which is why the total number of methods diverts from the number of included publications within this category.

For questionnaires, the studies employed virtual and real exposure measurements equally often with 49 cases of real greenspace usage and 50 times virtual greenspace being used. Physiological markers were equally split with 26 uses for both categories, while neurological indicators and cognitive testing were both similarly distributed. Qualitative measurements were gathered in combination with real greenspace four times and once combined with virtual greenspace (56). Behavior or face recognition were used as a mental health method four times with virtual greenspace in the studies with a predefined exposure. Three publications applied an assessment of both real and virtual greenspace which enabled them to include these two dimensions of exposure (153–155). Zhang et al. (153) used questionnaires as well as physiological markers, while the other two studies employed questionnaires.

## 4 Discussion

This scoping review represents the first comprehensive overview of the methods used for the assessment of greenspaces and mental health as well as their combinations. Accordingly, it provides an overview regarding the perspectives on greenspace and mental health research and identifies gaps as well as potentials for future research.

### 4.1 Identified patterns within the methodological combinations

A wide range of combinations regarding greenspace and mental health assessments was found, providing relevant insights regarding current use of methods (Figure 2). The results of this scoping review show distinctive patterns in the distribution of method combinations in the included studies, which represent certain perspectives regarding the influence of greenspace on mental health.

One of the most prevalent pairings identified was the combination of Questionnaires and GIS. This combination is considered to be a cost-effective method to investigate the interaction between a variety of mental health outcomes with different measurements of green space, mostly regarding the proximity to or abundance of greenspace (7, 26, 38). Additionally, these methods can be easily employed in order to research larger population sizes and longitudinal designs. Nevertheless, questionnaires mostly represent self-reported mental health measurements (156) and thus, only depict one dimension of mental health. GIS based studies (without additional information on exposure) cannot give evidence on the immediateness, visibility, quality, perceptions, usage and consciousness (2, 7). NDVI-based studies, in particular, have been criticized in the past as being too simplistic (157), land-use and land-cover data however can provide more information.

As pairings of methods are repeated, evidence is increasing regarding certain pathways which is an important aspect in order to gain insights into the effect of greenspace on mental health. Nevertheless, the repetition of methodological combinations can result in an imbalance of evidence and an incomplete assessment of use cases for greenspace. Less often utilized methodological combinations can provide insights into a more diverse set of greenspace influences on mental health. In line with our results, previous reviews identified the insufficient consideration of greenspace quality in the investigation of greenspace and mental health (1, 12). Methodological approaches to assess relevant qualities for the study population are for example expert assessments and especially qualitative research methods. Qualitative methods, such as interviews, can provide in-depth understanding of greenspace perceptions, consciousness, and reasons of greenspace uses (158, 159). Within the included studies, self-reported qualitative greenspace exposure measurements were examined less often than quantitative methods and almost exclusively combined with qualitative mental health measurements.

Several other reviews emphasize the relevance of individual behavior for the pathways restoration and instoration (7, 16). As individuals move around in their daily lives, their to greenspace changes. The accurate assessment of this exposure might require data on, e.g., time-activity patterns. For example, measurements of greenspace exposure in multiple locations which are visited in everyday life via EMA or SV could provide a much more realistic ecological and precise measurement of individual exposure of study populations (7, 160). However, they were rarely implemented within the included studies and mostly combined with questionnaires assessing mental health.

Some combinations were not found at all in the included studies. This might be due to limited methodological compatibility, such as predefined greenspace measurements in an intervention and ‘epidemiological measurements’. On the other hand, some method combinations which were not found in the review seem promising, such as greenspace measurements via ‘SV’ and the assessment of mental health with ‘physiological markers’. The employment of diverse methodological approaches in various combinations enhances the understanding of different facets and pathways, and improves the evidence in the field by providing a more comprehensive view. Also, several of the included studies used more than one combination of methods, as indicated by the greater number of combinations compared to the number of studies included. This combination of different methodological approaches can contribute to the diversification perspectives within this field of research.

### 4.2 Terminology of greenspace

Greenspace was described and defined in a variety of ways in the included studies. Nature, (natural) landscape, natural environment, greenspace, park, green infrastructure, and urban green were all applied to describe a number of greenspace types with little consistent uses or definitions in the reviewed papers. Greenness was another frequently used term, which can be utilized as a general term for vegetation quantity but not quality, type or accessibility (26). Furthermore, some papers investigated blue spaces without stating as such, which could only be determined by examining the corresponding results or supplementary material. This inconsistent and inaccurate use of greenspace definitions, as well as lacking descriptions of greenspace types limits the ability to compare study results.

This is especially true for publications regarding virtual greenspace which sometimes did not provide a description of the greenspace exposure. Browning et al. (161) emphasize the importance of selection and description of natural scenes in their review regarding the methodological choices in simulated landscapes. A detailed description of the used pictures or the accessibility via a cloud service enable the readers to understand what kind of greenspace is depicted (161). In order to ensure comparability of studies and the different evidence for greenspace on mental health a consistent usage of the terminology is necessary to enable an assessment of results. This is especially important for interdisciplinary research (Zerbe et al., submitted manuscript). The problem is not limited to empirical studies but includes concepts and theories regarding greenspace and human health. Many theoretical underpinnings do not differentiate between the types and scales of nature (Zerbe et al., submitted manuscript).

Literature informed definitions, such as provided by Taylor and Hochuli (20), can be an important source for a more consistent description of greenspaces. Furthermore, biological and environmental sciences have existing definitions of types and scales of nature, which can be the basis for finding a common language. In our review, it became evident that particularly the scale landscape is hardly described in detail and often kept quite vague as, for example, “countryside” which were, e.g., categorized as “rural greenspaces” (cf. Table 3; Zerbe et al., submitted manuscript). Moreover, different landscape types are often lumped together as “nature.”

### 4.3 Mental health outcomes and methods

The mental health outcomes identified in the included studies represent different layers of complexity and examine different facets of mental health. Outcomes such as mood or affect mostly assess the momentary emotional states of study participants. These offer an insight into specific pathways with which greenspace could influence the study population, but do not necessarily provide information regarding long-term mental health (21, 34). Other outcomes, e.g., manifested mental health disorders, describe a more complex assessment of the mental health status of study populations. However, these complex outcomes are the results of multifactorial influences. As such, it is difficult to determine the amount of influence greenspaces have on these outcomes.

In this scoping review, a wide range of methodologies regarding the assessment of mental health were found. To understand the complex pathways through which green spaces can affect mental health, multiple perspectives are needed to assess both physiologically observable and psychological processes (6, 38). Questionnaires were utilized most frequently which might be due to their cost-efficiency in assessing mental health (6). Regarding the quality of results, it has to be considered whether a validated instrument or a self-constructed questionnaire was used. A validated tool provides more consistent evidence for the mental health of study populations (162).

The psychopathology of mental illness is determined, among other processes, by a complex series of interactions between different biological mechanisms and various environmental factors (163). Using specific imaging and other techniques, neuroscience can provide additional information about the biological basis of mental illness and is therefore an important component in understanding and researching the identification of psychiatric biomarkers (163). Various structural or functional changes in the brain have been shown to be associated with a range of psychiatric disorders (163).

This scoping review identified a number of studies that investigated neuroscientific indicators in relation to exposure to the environment. However, when choosing brain structural and brain functional indicators as a proxy for mental illness, it is important to remember that at the current stage of research, there is still no clear mental disorder that can be diagnosed by a biomarker or based on a brain scan (164, 165). It is therefore relevant to recognize that neural indicators may have only limited explanatory power for actual disease patterns when considered in isolation. However, they can offer valuable insight into mechanistic pathways, depending on the aim of the research.

### 4.4 Real and virtual greenspace assessments

Research publications utilizing simulated natural elements are established within the research field of greenspace and mental health and their number is continuously increasing (161). Especially, virtual reality is frequently described as having the potential to provide an immersive natural environment, as well as the possibilities of digital activities inside simulated nature, when real natural environments are not available (161, 166).

Few of the included publications assessed real and virtual greenspaces in their greenspace measurements, although most did not compare the two dimensions. This is in line with results from a meta-analysis on this topic (167). Even fewer studies assessed the differences between natural and simulated environments. This comparison is especially important for the evaluation of potentials as well as limitations of virtual greenspaces. A previous meta-analysis showed virtual greenspace to have a smaller positive influence on positive affect in comparison to real natural greenspace (167). This might be due to the possibly restricted number of pathways virtual greenspace exposure operates on, as it mainly includes visual or audio stimuli or a combination of the two pathways (168). Therefore, it can be presumed that the benefit of actual greenspace, with its potential for physical activities, social connection, sense of place and multisensory experiences, is greater than the mostly visual or auditory activation of virtual greenspace (167). Within the last years, there is also evidence that the appreciation regarding real greenspaces has increased due to the COVID 19-pandemic (35, 36).

Also, the increased use of virtual environments might enhance the extinction of experience, which has been linked to less positive attitudes toward nature conservation and also less benefits from interacting with nature as more nature contact is connected to more nature connectedness and, in turn, to more happiness (169, 170). Simulated greenspaces cannot fully replace real greenspace but the continued comparison is necessary to evaluate the most suitable applications for technologies such as virtual reality.

### 4.5 Identified research gaps and recommendations for future research

This section synthesizes the findings within this review. The combinations used to investigate the relation of greenspace and mental health within the included studies represent particular perspectives on greenspace or mental health (26). While every pairing provides valuable insight into the connection between greenspace and mental health, the pathways of interaction are diverse (7) and as such have to be evaluated accordingly. There is a need for a variety of methods in order to offer different perspectives on exposures as well as outcomes (14, 160), since every isolated combination has its potentials as well as its weaknesses. High quality assessments of the potential impact greenspace might have on mental health provide valuable insights into the planning of future greenspaces and the urban environment (6, 7, 21).

Through this scoping review a lack of real mixed method approaches was identified as qualitative methodologies were most often utilized for both greenspace as well as mental health. Combinations of qualitative with quantitative measures such as interviews and GPS based data could provide a more detailed view into the effect of greenspace exposure and mental health outcomes (151, 171). The measurement of daily green space exposure using methods such as EMA or SV is also still rarely used and almost exclusively combined with questionnaires. Individual studies already implement other mental health assessments, such as epidemiological measurements or neurological indicators (66, 152), but these potentials need to be investigated further. The identification of these gaps can provide directions regarding future research as it shows ways off the beaten paths. More publications combining and utilizing different methods are needed, as their findings offer important evidence for informed decision-making regarding the future development of greenspaces and their potentials for mental health.

Interdisciplinary research efforts are another opportunity to broaden the perspectives on this complex research field. Collins et al. (12) underline the importance of considering greenspace quality from both, a human and an ecological perspective in order to assess the ecosystem services provided by greenspace. The interdisciplinary combination of different perspectives and approaches can improve the building of hypothesis as well as the research process regarding the mechanisms and pathways (14). One aspect of this collaboration should be the identification of suitable definitions of greenspace, as well as the adequate description of the utilized greenspaces. Especially regarding virtual exposure, the description of greenspace is needed in order to investigate these technologies potentials, shortcomings, as well as potential risks within future research. The usage of reporting frameworks for exposures and outcomes, e.g., PRIGSHARE for satellite based greenspace assessments (172) can improve the comprehensibility and thus the quality of evidence.

### 4.6 Potentials and limitations of this scoping review

This scoping review featured an extensive literature review in four electronic databases and provides a broad overview over the scientific literature. The wide range of included studies with inclusion of experimental, population based and qualitative studies facilitates the insights into this research field is a notable strength of this review. Due to the vast number of publications, the wide range of applicable studies as well as the aim to reflect the current methods used, only studies published between 2017 and 2022 were included in the review. Despite the extended search strategy, there might be some relevant publications not found in the search process and accordingly not included in the review. This could result in studies employing unique combinations not being found although a broad search was used to counteract this possibility. Some overlap exists in the categories employed in this review. For example, studies using a predefined greenspace exposure often involve an expert assessment of the greenspace in order to find suitable study sites. This might take away from the category expert assessment, especially in combination with physiological markers, cognitive testing and neurological indicators. Rural greenspaces while not being excluded within the screening process were also not explicitly integrated in the search strategy of this review, which could result in studies regarding mental health and rural greenspace being underrepresented in this review. The information from the included studies were manually extracted in this review, as an automated process would have likely led to skewed results, due to inconsistent terminology in the studies. However, this inconsistency could also bias the search and screening steps within the manual extraction of results.

## 5 Conclusion

This review found that a considerable number of studies researched the effect of greenspace and mental health with similar methodological combinations. While the repetition of methodologies can increase the evidence regarding certain aspects within this field of research, due to the complexity of this connection, it is necessary to explore less-frequently used combinations and study designs, such as mixed method studies. New technologies such as virtual or augmented reality need to be assessed regarding their specific benefit for populations to complement real greenspaces. Broader perspectives can enable researchers, urban planners and decision-makers to gain a deeper understanding of which greenspace provide certain mental health benefits and how these greenspaces can be implemented in future human habitats. This necessitates a common understanding of greenspace types and scales as well as mental health outcomes. As studies often utilize different definitions of greenspace or even fail to provide an accurate description, the comparability of evidence is limited. Similarly, mental wellbeing, emotional states and psychological illnesses are distinct outcomes and may require different methods to assess accurately. Interdisciplinary research collaborations can enhance the quality of evidence as existing, precise definitions from various fields of research can enable a deeper understanding of the connection between greenspace and mental health. Research methods from a range of scientific fields such as ecology, public health, psychology, medicine or urban planning can provide potentials leading to more diverse and profound evidence.

## Data availability statement

The original contributions presented in the study are included in the article/Supplementary material, further inquiries can be directed to the corresponding author.

## Author contributions

JF: Conceptualization, Data curation, Methodology, Writing – original draft, Writing – review & editing, Visualization. H-LS: Conceptualization, Data curation, Methodology, Writing – original draft, Writing – review & editing. BS: Conceptualization, Data curation, Methodology, Writing – original draft, Writing – review & editing. SLL: Writing – review & editing, Data curation. SZ: Funding acquisition, Writing – review & editing. CH: Funding acquisition, Writing – review & editing. TM: Conceptualization, Data curation, Funding acquisition, Writing – review & editing.
