# Supplementary material for: Current methodologies of greenspace exposure and mental health research—a scoping review
Source: Front Public Health. 2024 Mar 5;12:1360134. doi: 10.3389/fpubh.2024.1360134 (PMC10951718; doi:10.3389/fpubh.2024.1360134)
Supplement: Supplementary file 1 [file Table_1.DOCX]

Table 1: Search terms used for screening process via the PEO-Framework with numbered references to data bases

| Population | Exposure | Outcome |
| --- | --- | --- |
| Urban* | green space* | mental health*  mental health ^1, 2, 3^ |
| City*  Cities ^1, 2^ | greenspace* | cognitive function* |
| Metropolitan* | Green infrastructure* | cognition*  cognition ^1, 2, 3^ |
| Town*  Towns ^3^ | Park  Parks  Parks, Recreational ^1^  recreational park ^2^ | psychological health* |
| residential* | Vegetation*  Vegetation ^2^ | restoration* |
| agglomeration* | Tree*  Trees ^1^  Tree ^2^ | rehabilitation*  rehabilitation ^1, 2, 3^ |
| municipality* | green roof* | healing*  healing ^2^ |
|  | ecology*  ecology ^1, 2, 3^ | recovery* |
|  | garden*  gardens ^1^ | mood*  mood ^2^ |
|  | wilderness*  wilderness ^2^ | satisfaction*  personal satisfaction ^1^  satisfaction ^2, 3^ |
|  | forest*  forests ^1^  forest ^2^ | happiness*  happiness ^1, 2, 3^ |
|  | agriculture*  agriculture ^1,2^ | well-being* |
|  | fields* | wellbeing*  wellbeing ^2^ |
|  | grass*  grass ^2^ | mental disorder*  mental disorder^1^  mental disorders ^3^  mental disease ^2^ |
|  | woodland* | stress*  stress, psychological^1^  mental stress ^2^  stress ^3^ |
|  | greenway* | psychological disorder* |
|  | greenery* |  |
|  | plants*  plants ^1^  plant ^2^ |  |
|  | green belt* |  |
|  | natural environment* |  |
|  | ecosystem*  ecosystem ^1,2^ |  |
|  | natural space* |  |
|  | nature*  nature ^1, 3^ |  |
|  | landscape*  landscape ^2,^ |  |
|  | nature based solution* |  |
|  | horticulture*  horticulture ^1,2^ |  |
|  | biodiversity*  biodiversity ^1,2^ |  |

Specific search terms used within the individual data bases:

^1:^ Pubmed

^2:^ Embase

^3:^ PsycInfo

^4:^ Web of Science
